# Phosphorylation of PPARγ at Ser84 promotes glycolysis and cell proliferation in hepatocellular carcinoma by targeting PFKFB4

**DOI:** 10.18632/oncotarget.12764

**Published:** 2016-10-19

**Authors:** Yuxin Shu, Yan Lu, Xiaojuan Pang, Wei Zheng, Yahong Huang, Jiahong Li, Jianguo Ji, Can Zhang, Pingping Shen

**Affiliations:** ^1^ State Key Laboratory of Pharmaceutical Biotechnology and Model Animal Research Center (MARC), Nanjing University, Nanjing 210023, China; ^2^ Center of Drug Discovery, State Key Laboratory of Natural Medicines, China Pharmaceutical University, Nanjing 210009, PR China; ^3^ The State Key Laboratory of Protein and Plant Gene Research, College of Life Sciences, Peking University, Beijing 100871, China; ^4^ Institute of System Biomedicine, School of Basic Medical Sciences, Peking University, Beijing 100191, China

**Keywords:** phosphorylation, PPARγ, PFKFB4, hepatocellular carcinoma, glycolysis

## Abstract

Peroxisome proliferator-activating receptor γ (PPARγ), a transcription factor, is involved in many important biological processes, including cell terminal differentiation, survival and apoptosis. However, the role of PPARγ, which regulates tumour promoter and oncogene expression, is not well understood in hepatocellular carcinoma (HCC). In the present study, based on evidence from clinical samples that phosphorylation of PPARγ at Ser84 is up-regulated in human liver tumours, we confirmed that phosphorylation of PPARγ was also significantly increased in an HCC mouse model and was increased by Mitogen-activated protein kinase (MEK)/ Extracellular-signal-regulated kinases (ERK) kinase. Next, we performed an RNA microarray analysis, and our data indicated that dephosphorylation of PPARγ at Ser84 affects the expression of glycolysis-related genes and pro-proliferation genes, which supposedly promote proliferation of HCC cells. Using a chromatin immunoprecipitation (ChIP) assay, we demonstrated that the observed PPARγ-mediated induction of 6-phosphofructo-2-kinase/fructose-2,6-biphosphatase 4 (PFKFB4) expression was directly modulated by the transcriptional activity of its promoter. Furthermore, using knockdown of PFKFB4, we elucidated that the stimulation of PPARγ phosphorylation on glycolysis and proliferation in HCC is dependent on PFKFB4. Together, these findings extend our understanding of how liver tumour cells reprogram their glycolytic pathways by post-translational modification of specific transcription factors and lay a foundation for the screening of new targets for the treatment of HCC.

## INTRODUCTION

Hepatocellular carcinoma (HCC) is currently the sixth most common cancer worldwide and remains an extremely complex condition with a poor prognosis [1, 2]. HCC development is a multistep and long-term process and is primarily associated with hepatitis B virus or hepatitis C virus. Other risk factors include alcoholic liver cirrhosis, nonalcoholic steatohepatitis, intake of aflatoxin-B1-contaminated food and metabolic disorder [3, 4]. The malignant transformation of hepatocytes is tightly correlated with genetic changes [5] and subsequently aberrant regulation of multiple signalling cascades [6–9]. Notably, the alterations to transcription factor function confer the specific advantages necessary for hepatocyte transformation and thereafter the fate of hepatocarcinoma cells [10, 11].

Peroxisome proliferator-activating receptor γ (PPARγ) is an isoform of the PPAR nuclear receptor family that functions as a transcription factor. Numerous studies have proven that PPARγ is involved in many important biological processes, including cell terminal differentiation, survival and apoptosis, and thereby plays an essential role in regulating adipogenesis, inflammation, tumourigenesis and metastasis etc.[12]. In fact, some research has supported the idea that PPARγ can act as a tumour suppressor or tumour promoter, depending on the tumour type and development stage [13]. Most interestingly, there have been encouraging reports revealing that PPARγ activation prevents cancer in tissues such as colon, breast, prostate, lung and liver [14], justifying that PPARγ agonists may be useful in hepatocellular carcinoma therapy [15]. In diabetic patients, the use of PPARγ agonists (pioglitazone and rosiglitazone) is associated with decreased liver cancer incidence [16]. All of the evidence above stimulated studies on the role of PPARγ in HCC and the possibility of targeting it.

**Figure 1:**
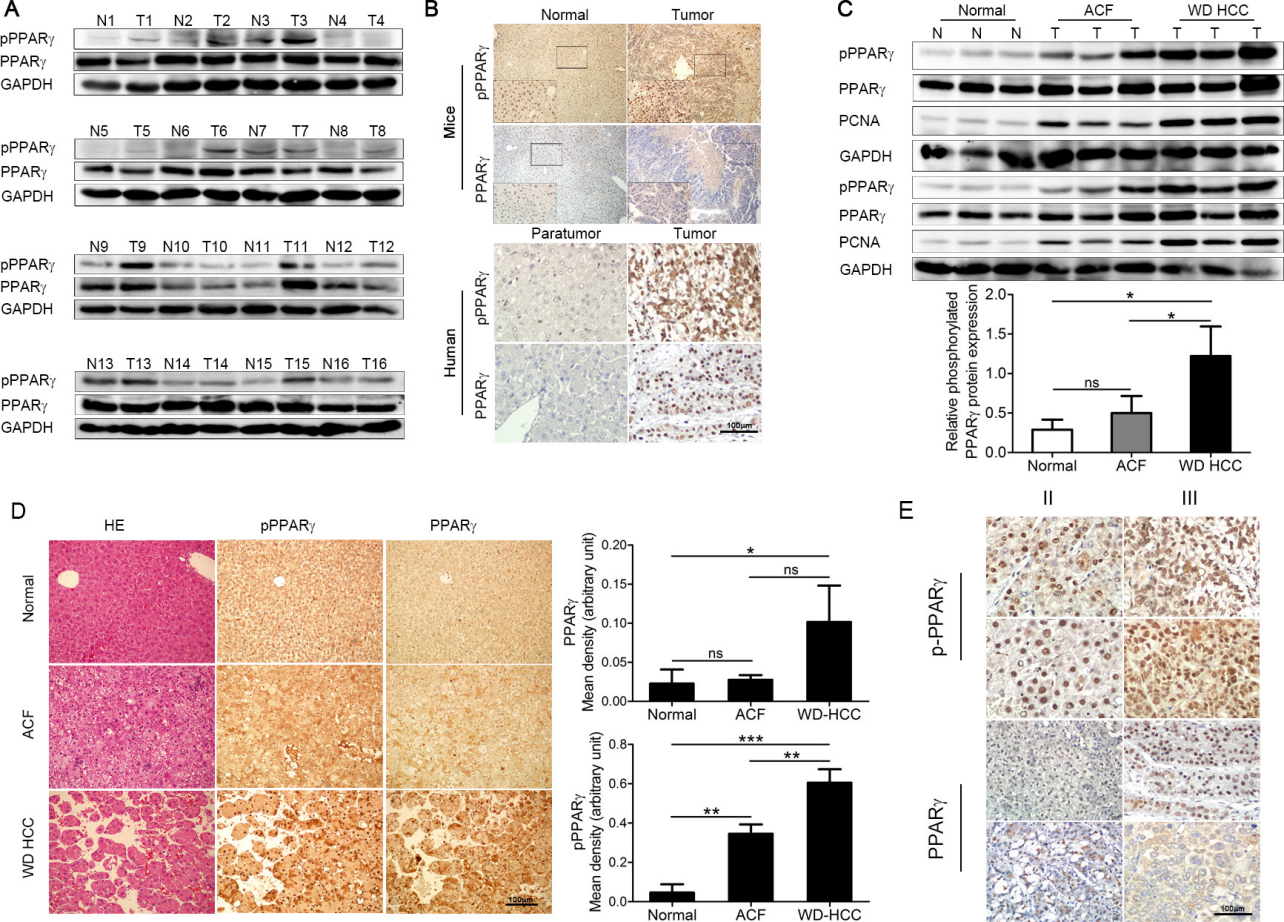
PPARγ phosphorylation at Ser82/Ser84 is up-regulated in HCC (**A**) Western blot of PPARγ and phosphorylated PPARγ in normal liver tissue and tumour of DEN-induced mouse model of HCC. (**B**) Immunostaining of PPARγ and phosphorylated PPARγ in normal/para-tumour live tissue and liver tumour of mice (upper panel) and human (lower panel). (**C**) Western blotting analysis of PPARγ, phosphorylated PPARγ and PCNA in normal mouse liver tissue, ACF (abnormal cell foci), WD HCC (well-differentiated HCC) (upper panel). Quantification of relative phosphorylated PPARγ expression shown in C (lower panel). (**D**) HE of PPARγ and phosphorylated PPARγ in normal mouse liver, ACF, WD HCC. Quantification of PPARγ and phosphorylated PPARγ expression in D by imageJ software. (**E**) Immunostaining of PPARγ, phosphorylated PPARγ in human phase II and phase III liver tumour.

PPARγ can be phosphorylated at Ser112 (Ser82 in PPARg1) and Ser273 by mitogen-activated protein kinases (MAPKs) and cyclin-dependent kinases, respectively [17–19]. Normally, PPARγ phosphorylation at Ser112 represses its transcriptional activity by inhibiting ligand binding and altering cofactor recruitment, whereas the mutation of the phosphorylation site by changing Ser112 into alanine leads to increased transcriptional activity [20]. However, in contrast to previous studies showing that the phosphorylation of Ser112 was inhibitory, cdk7-mediated phosphorylation stimulates PPAR-γ transcriptional activity, suggesting that the phosphorylation of PPARγ may stimulate its transcriptional activity in certain tissues (e.g., BAT) or under certain conditions [21]. Furthermore, covalent modification of PPARγ phosphorylation at Ser112 is a major regulator of the balance between cell growth and differentiation in the adipose cell lineage [22]. We have previously demonstrated the pro-proliferative effects of PPARγ phosphorylation at Ser84 (Ser112 in mouse) in human fibrosarcoma cells [23]. Inspired by the evidence that phosphorylation of PPARγ at Ser84 was significantly up-regulated in liver cancer tissue (Figure 1), we are interested in determining the involvement of PPARγ phosphorylation during HCC development.

Liver tumour cells reprogram their metabolic pathways to meet their needs during the process of tumour progression. The best-characterized metabolic phenotype observed in tumour cells is the Warburg effect, which is a shift from ATP generation through oxidative phosphorylation to ATP generation through glycolysis, even under normal oxygen concentrations [24]. The enzymes within the glycolysis metabolic pathways have been shown to be essential for the growth and survival of cancer cells, such as pyruvate kinase [25], hexokinase [26] and phosphofructokinase [27]. Among these key glycolytic enzymes, 6-phosphofructo-2-kinase/fructose-2,6-biphosphatase 4 (PFKFB4), an isoform of the glycolytic enzyme phosphofructokinase 2 (PFK2), modulates the intracellular concentration of the allosteric glycolytic activator, fructose-2,6-biphosphate (F2,6BP), which is a key regulator of glycolysis [28]. PFKFB4 has been shown to be expressed in multiple organs and to be overexpressed in human tumours, indicating a potential role in cancer development and/or progression [29, 30]. Until now, few studies have demonstrated that PFKFB4 is induced by hypoxia and required for the survival and growth of several cancer cell lines [30, 31]. Although PFKFB4 has proven to be a target for anti-tumour drug development [32], its regulation and activation *in vivo* is still not clear.

In the present study, we found that phosphorylation of PPARγ at Ser82/Ser84 was up-regulated in mouse (Ser82) and human (Ser84) liver tumours and is increased by MEK/ERK kinase. Next, our data indicated that phosphorylation of PPARγ at Ser84 stimulated the expression of glycolysis-related genes and pro-proliferation genes. We also demonstrated that the observed PPARγ-mediated induction of PFKFB4 expression was directly increased by the transcriptional activity of its promoter. Together, these findings extend our understanding of how liver tumour cells reprogram their glycolytic pathways by post-translational modification of specific transcription factors and lay a foundation for the screening of new targets for the treatment of HCC.

## RESULTS

### PPARγ was phosphorylated at Ser82/Ser84 in mouse and human liver tumours

A diethylnitrosamine (DEN) mouse model of HCC was established, and mice were randomized to control. Sixteen pairs of liver samples from normal (N) mice or tumour-bearing (T) mice were collected to detect the phosphorylated and total PPARγ by western blot (Figure 1A). According to the quantification of western blot bands (Supplementary Figure S1A), the level of PPARγ phosphorylation in the liver in tumour mice was 2.9 times higher than that in normal mice. Similarly, the immunostaining of phosphorylated PPARγ and total PPARγ in live tissue from normal/tumour mice (up panel of Figure 1B) also showed high levels of phosphorylated PPARγ in tumours. Furthermore, PPARγ was obviously located in the nucleus of hepatocytes (Figure 1B), which might be related with the major function of PPARγ as a transcription factor.

Next, we focused on the development of an HCC mouse model, which was treated with DEN for 10, 11 or 12 months. According to the tumour region, we collected different samples of liver tissue, such as abnormal cell foci (ACF) in liver and well-differentiated HCC (WD-HCC). Proliferating cell nuclear antigen (PCNA), evaluated as a marker of cell proliferation, was elevated (Figure 1C) in ACF and WD-HCC samples, which verified the higher cell proliferation in these abnormal liver tissues. Phosphorylated and total PPARγ were measured by western blot (Figure 1C) and immunostaining (Figure 1D) in normal liver tissue, ACF and WD of HCC. Quantification of those experiments (Figure 1D) indicated that levels of phosphorylated PPARγ in WD-HCC were significantly higher than those in normal and ACF liver tissue, but the difference in total PPARγ expression between normal and ACF tissue or between ACF and WD-HCC was not significant (*P* > 0.05). The huge difference of PPARγ expression in WD-SCC might be caused by heterogeneity of tumor cells (Figure 1D).

The human HCC liver samples were used for further verification. In the sections of human HCC analysed by immunostaining, phosphorylated PPARγ in the tumour was increased compared with that in the paratumour (down panel of Figure 1B). Furthermore, the level of PPARγ phosphorylation (Figure 1E) in phase III human HCC had a 2.7-fold increase compared with that in phase II. After that, we also compared PPARγ phosphorylation in Ser273 between the normal and tumour tissue from the mice liver and between phase II and III tumour from human liver (Supplementary Figure S1E). And we found that there were no significant difference, which exclude that, the site of Ser273 can be the main way to regulate PPARγ here. All results above indicate that PPARγ phosphorylation is associated with the generation and development of HCC.

### MEK/ERK phosphorylates PPARγ and increases the proliferation of HCC

**Figure 2:**
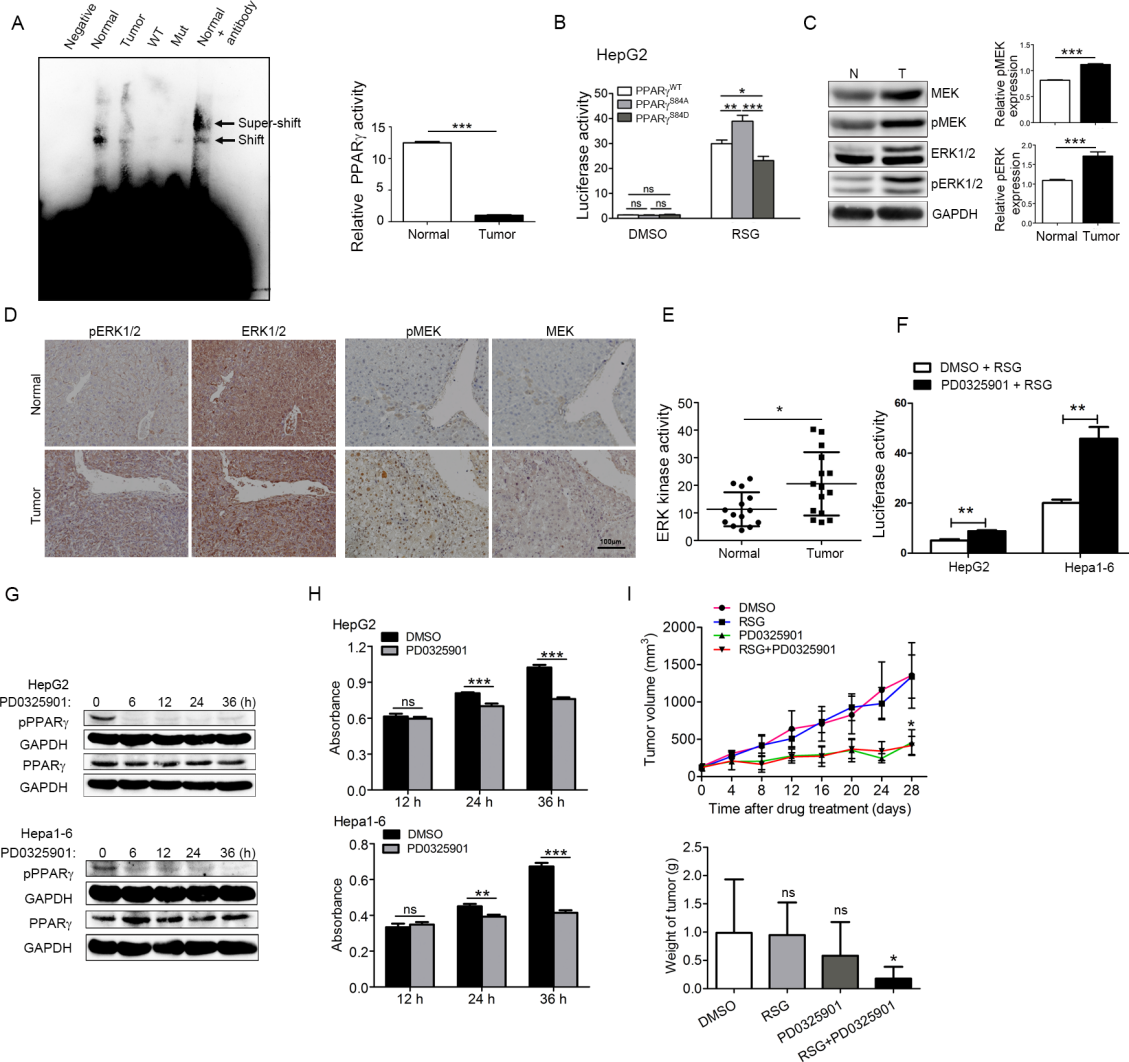
Phosphorylation of PPARγ up-regulated by MEK/ERK kinase increases the proliferation of HCC (**A**) PPARγ transcriptional activity in normal liver and hepatocyte carcinoma measured by EMSA; Normal, Normal liver tissue; tumour, tumour liver tissue; Negative, without oligonucleotides control; Mut, Biotin end-Labeled mutant; WT, cold competitor. (**B**) PPARγ transcriptional activity in hepG2 cell overexpressing PPARγ^WT^, PPARγ^SA^ and PPARγ^SD^ measured by luciferase reporter with/without RSG. (**C**) Phosphorylation level of ERK and MEK in tumour and normal liver tissue by western blot. (**D**) Phosphorylation level of ERK and MEK in tumour and normal liver tissue by immunostaining. (**E**) ERK kinase activity in tumour and normal liver tissue is measured by ERK kinase activity kits. (**F**) PPARγ transcriptional activity was measured in HepG2 and Hepa1-6 treated with PD0325901 for 12 hrs using luciferase reporter gene assay. (**G**) the expression of p-PPARγ and PPARγ in HepG2 and Hepa1-6 treated with PD325901 for 0, 6, 12, 24, and 36 hrs separately. (**H**) Proliferation of HepG2 and Hepa1-6 treated with or without PD0325901. (**I**) Tumour growth curve of mice treated with DMSO, RSG only, PD0325901 only, or RGS+ PD0325901.

Our previous study suggested that phosphorylation of PPARγ at Ser84 attenuated PPARγ transcriptional activity [23]. To further understand the characteristics of PPARγ in HCC, we used EMSA to explore its transcriptional activity. As shown in Figure 2A, PPARγ binds PPRE more effectively in normal liver tissue than in HCC liver tissue, which indicates significantly (*P* < 0.001) down-regulated (12.4-fold) transcriptional activity in mouse HCC, in concert with the up-regulated phosphorylation of PPARγ. To verify that PPARγ phosphorylation suppresses PPARγ transcriptional activity, we established stable clones of HepG2 overexpressing PPARγ^WT^, PPARγ^SA^ (non-phosphorylation mutant, Ala instead of Ser84) or PPARγ^SD^ (phosphorylation mutant, Glu instead of Ser84). As expected, compared to the WT, PPARγ transcriptional activity in HepG2 overexpressing PPARγ^SA^ was up-regulated, but that in PPARγ^SD^ was down-regulated (Figure 2B), implicating that phosphorylation of PPARγ at Ser84 attenuated PPARγ transcriptional activity.

Several studies have shown that MEK/ERK is an up-stream kinase that phosphorylates PPARγ at Ser112 [34], which suggests potentially increased activity of MEK/ERK in HCC. Therefore, we first analysed the phosphorylation level of ERK and MEK in tumour and normal liver tissue by western blot to determine the kinase activity of ERK and MEK. The quantification for western blot showed that 56% of ERK and 36% of MEK phosphorylation was up-regulated in HCC compared with the normal mice (Figure 2C). The similar results obtained by immunostaining in the sections of mouse liver tissue suggest that the phosphorylation levels of ERK and MEK in the liver of HCC mice were significantly higher than those of normal mice (Figure 2D). Consistent with the phosphorylation level of ERK/MEK analysed by immunostaining and western blot, the ERK kinase *in vitro* assay demonstrated that ERK activity is apparently increasing in the liver of HCC mice (Figure 2E).

The MEK inhibitor PD0325901 [35] was used to block the up-stream ERK kinase to inhibit phosphorylation of PPARγ. As the results show in Figure 2G and Supplementary Figure S2B, when the HCC cell lines, such as HepG2 and Hepa1-6, were treated with PD0325901 for more than 6 hrs, phosphorylation of PPARγ obviously decreased. As expected, treated with PD0325901, PPARγ transcriptional activity accordingly increased significantly (*P* < 0.01) in HepG2 and Hepa1-6, not in SMMC7721 or Hep3B, according to a luciferase reporter gene assay (Figure 2F and Supplementary Figure S2C).

In agreement with a previous report [36], after 24 hrs treatment with PD0325901, the proliferation of HepG2 and Hepa1-6 (Figure 2H) were inhibited significantly (Figure 2H). But the proliferation of SMMC7721 and Hep3B was not significantly decreased by PD0325901, indicating that cell proliferation may be related with the PPARγ transcriptional activity (Supplementary Figure S2D). Consistent with the *in vitro* study, the *in vivo* results showed that the volume and weight of tumours in DEN-induced HCC mice were inhibited by PD0325901. Furthermore, the inhibitory effects on tumour growth under treatment with PD0325901 together with rosiglitazone (RSG), an agonist of PPARγ, were better than with PD0325901 alone. [37] (Figure 2H, 2I and Supplementary Figure S2E). Taken together, our findings strongly support that down-regulation of PPARγ phosphorylation or transcriptional activity can promote the proliferation of HCC *in vivo* and *in vitro*, which indicates that PPARγ phosphorylation is required for HCC proliferation.

### Phosphorylation of PPARγ promotes proliferation of HCC

**Figure 3:**
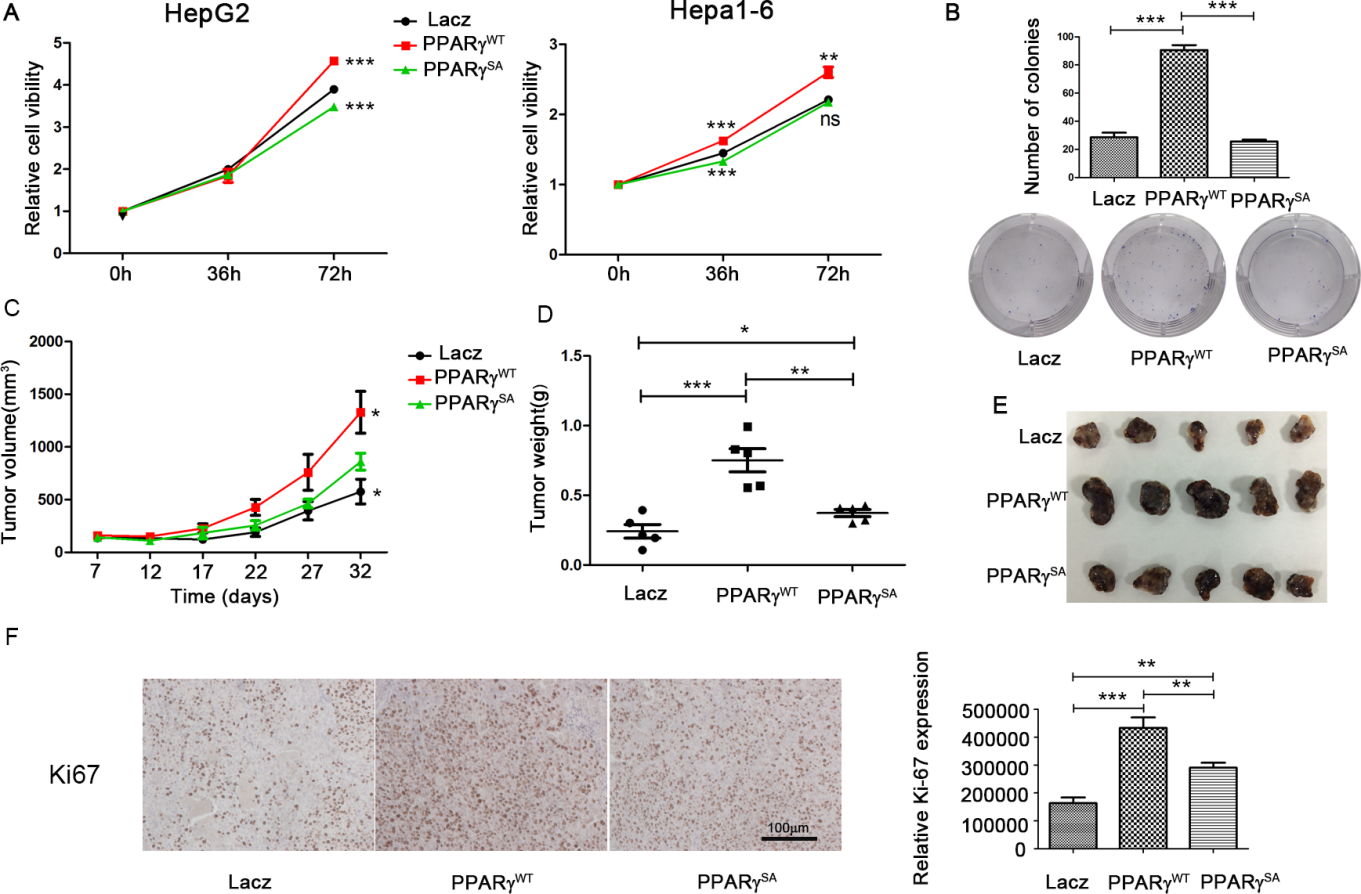
Phosphorylation of PPARγ promotes the proliferation of HCC (**A**) The proliferation of cell lines (HepG2 and Hepa1-6) stably overexpressing PPARγWT, PPARγS112A or lacZ tested by CCK8 kits. (**B**) Methylthiazol tetrazolium and clonogenicity assay in these cell clones. (**C**) Volume of tumours in nude mice implanted with HepG2 stably overexpressing PPARγWT, PPARγS112A or lacZ. (**D**) Weight of tumours in nude mice implanted with HepG2 stably overexpressing PPARγWT, PPARγS112A or lacZ. (**E**) Tumours in nude mice implanted with HepG2 stably overexpressing PPARγWT, PPARγS112A or lacZ. (**F**) Expression of Ki67 in these tumours by IHC analysis.

To investigate the effect of PPARγ phosphorylation on the proliferation of HCC, we used stable clones of HepG2 and Hepa1-6 cell lines overexpressing PPARγ^WT^, PPARγ^SA^ or lacZ separately (Figure 3A). As shown in Figure 3A, after 72 hrs incubation, both cell lines overexpressing PPARγ^WT^ grew faster than those overexpressing PPARγ^SA^ and LacZ. Moreover, HepG2 overexpressing non-phosphorylated PPARγ (PPARγ^SA^) grew slower than cells overexpressing PPARγ^WT^ and LacZ, which supports the role of PPARγ phosphorylation in the promotion of HCC. Accordingly, in the colony formation assay, the cells with PPARγ^WT^ formed more than twice the number of colonies than the cells with PPARγ^SA^ and LacZ (Figure 3B). According to the PI/annexin V analysis, there is no difference in the proportion of apoptotic cells among HepG2 overexpressing PPARγ^WT^, PPARγ^SA^ and lacZ (Supplementary Figure S3A) so that the PPARγ phosphorylation on the apoptosis can be excluded here.

To assess the *in vivo* proliferation of the stable transfection clones of HepG2 with PPARγ^WT^, PPARγ^SA^ or lacZ, these cell clones were implanted into the nude mice separately, and obvious tumours formed after 7 days. The largest tumour volume (Figure 3C) and weight (Figure 3D) were observed in the tumours with PPARγ^WT^. Similarly, the volume and weight of the tumours with non-phosphorylated PPARγ (PPARγ^SA^) were smaller compared to the PPARγ^WT^ tumours but were larger compared to the LacZ tumours (Figure 3C, 3D and 3E). Moreover, the expression of Ki-67 protein, which is strictly associated with cell proliferation, was increased significantly in PPARγ^WT^ tumour tissue compared to the tissues with PPARγ^SA^ (1.5-fold) and LacZ (2.6-fold) according to immunostaining analysis (Figure 3F). All these *ex/in vivo* results indicate that phosphorylation of PPARγ promotes the growth and survival of HCC.

### PFKFB4 is the target gene of PPARγ

**Figure 4:**
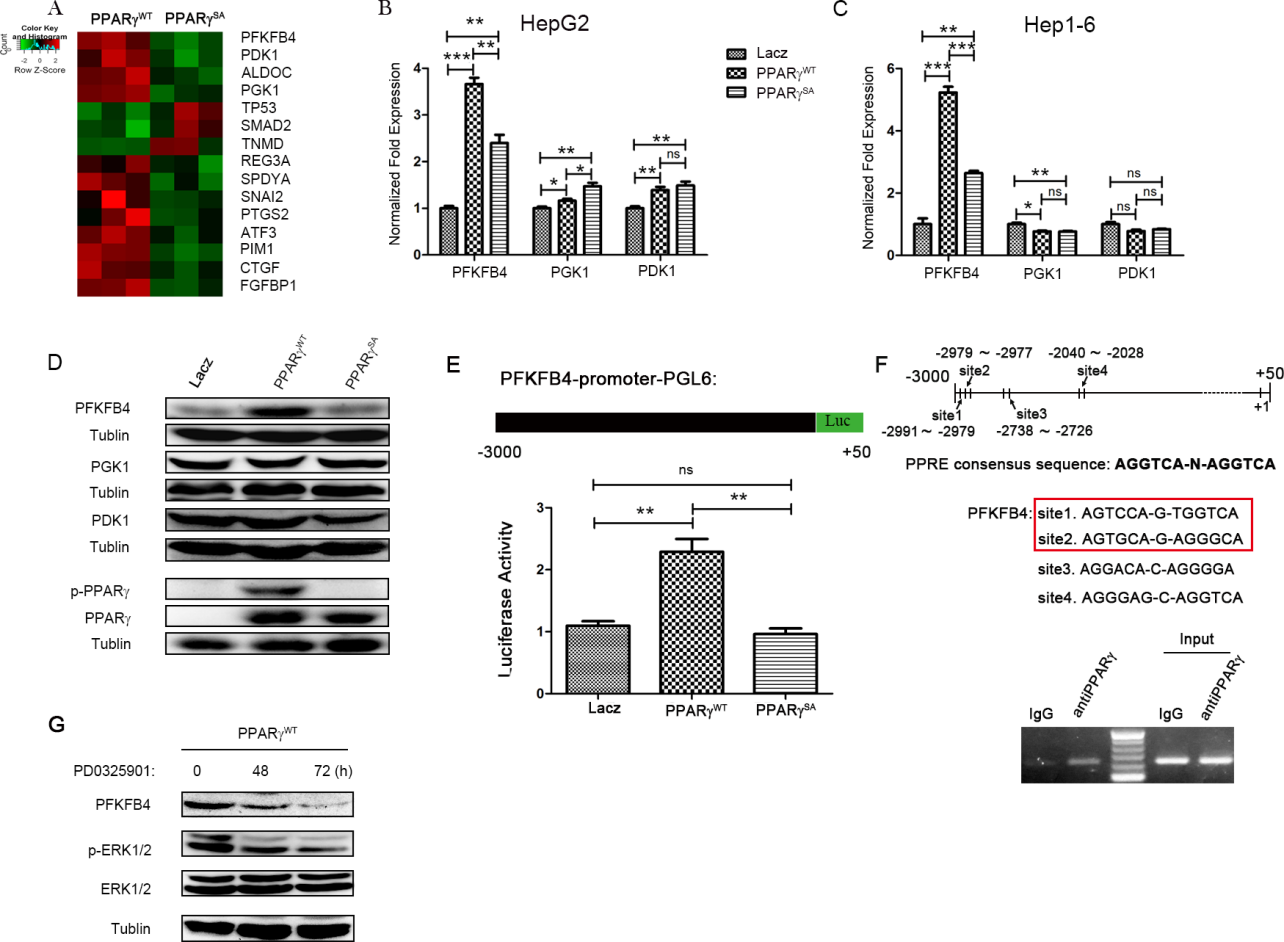
PFKFB4 is a target gene of PPARγ gene-expression data from HepG2 cells transfected with PPARγ^WT^ or PPARγ^SA^ (**A**) Each row represents an individual gene, and each column represents a transfected HepG2 cell sample. In the matrix, red and green reflect relatively high and low expression levels of genes, respectively, as indicated in the scale bar (a log2-transformed scale). (**B** and **C**) qRT-PCR experiments with mRNAs from the indicated cancer cell lines after transfection with lacZ, PPARγ^WT^ or PPARγ^SA^. (**D**) Western blotting analysis of the three glycolysis enzymes, p-PPARγ & PPARγ in HepG2 cells transfected with indicated plasmid. (**E**) A pGL3 luciferase reporter plasmid was constructed with the promoter of PFKFB4. The activity of PFKFB4 promoter was detected by luciferase reporter from HepG2 cells transfected with lacZ, PPARγ^WT^ or PPARγ^SA^. (**F**) Prediction by PPRE research, four sequences in the human PFKFB4 promoter (−3000~+50) displayed significant homology with the PPARγ recognition motif. HepG2 were harvested for ChIP analysis with an anti-PPARγ antibody or preimmune IgG using site1 and site2 chip primers. Input chromatin was diluted to 1:1000. All PCR products were resolved by 2% agarose electrophoresis. (**G**) Western blotting analysis of phosphorylation level of ERK and PFKFB4 expression in PPARγ^WT^ (HepG2) cell line treated with PD325901 for 0, 48 and 72 hrs separately. (*P < 0.05; **P < 0.01; ***P < 0.005; by Student *t* test).

To investigate how PPARγ phosphorylation regulates its functions, the differential gene expression between PPARγ^WT^ and PPARγ^SA^ hepG2 cells was analysed by gene chip (*P* < 0.01). The resulting heat map (Figure 4A) demonstrated that the expression of genes related to glycolysis and pro-proliferation were down-regulated in the PPARγ^SA^ cell line, which indicates that de-phosphorylation of PPARγ at Ser84 decreases the expression of glycolysis-related genes and pro-proliferation genes. The transcription factor hypoxia-inducible factor-1 (HIF-1) pathway is responsible for the induction of genes that facilitate adaptation and survival of cells and mainly involved in glycolysis and proliferation. However, there was no difference in the expression of HIF-1a protein from HepG2 with PPARγ^WT^, PPARγ^SA^ or lacZ, which lead us to focus on glycolysis-related genes here (Supplementary Figure S4C). Therefore, differential expression of glycolysis-related genes induced by different levels of PPARγ phosphorylation may be one of the reasons that PPARγ phosphorylation at Ser84 promotes the growth and survival of HCC.

As expected, de-phosphorylation of PPARγ can cause the down-regulation of genes that are involved in cell growth (e.g., PTGS2, ATF3, CTGF, and SPDYA). Surprisingly, a large number of the genes (e.g., PFKFB4, PGK1 and PDK1) that are directly involved in glycolysis are also down-regulated by de-phosphorylation of PPARγ, strongly suggesting a potential role for PPARγ in regulating glucose metabolism (Figure 4A and Supplementary Figure S4B). To verify the results of the gene chip analysis, we utilized qPCR to measure the expression of genes with significant differentiation (> 200%), such as PFKFB4, PGK1 and PDK1, which play important roles in glycolysis and Warburg's effect [38]. As shown in Figure 4B and 4C, expression of PFKFB4 in PPARγ^SA^ cells was less than that in PPARγ^WT^ cell lines, such as HepG2 (0.65-fold) and Hep1-6 (0.5-fold), suggesting that phosphorylation of PPARγ at Ser84 might increase the transcription of PFKFB4. However, expression of PFKFB4 was increased in PPARγ^WT^ and PPARγ^SA^ cells compared to the lacZ-control, possibly because of the overexpression of PPARγ in both the PPARγ^WT^ and PPARγ^SA^ cell lines. Consistent with the alteration of PFKFB4 mRNA expression, the up-regulated expression of PFKFB4 protein by PPARγ overexpression (PPARγ^WT^) disappeared in the PPARγ^SA^ cell line where PPARγ phosphorylation at Ser84 was accordingly blocked (Figure 4D). In contrast, the expression of the other two proteins, PGK1 and PDK1, were not changed significantly (Figure 4D). On the other hand, PD0325901 can suppress PFKFB4 expression in PPARγ^WT^ stable clone (Figure 4G), which might offer an evidence for the MEK/ERK-PPARγ-PFKFB4 pathway in HCC formation. Therefore, we were interested in the relationship between PFKFB4 and PPARγ because no previous reports have clarified this.

To identify PPARγ as the candidate transcription factor regulating PFKFB4 expression, a PFKFB4 promoter (−3000~+50)-luciferase reporter gene assay was used (Figure 4E). As the data in Figure 4F show, when PPARγ was overexpressed, the signal from the luciferase reporter gene system was increased compared to the lacZ control (more than 1.5-fold), which indicates that the promoter of PFKFB4 was activated by PPARγ accordingly. Furthermore, the de-phosphorylation of PPARγ at Ser84 reduced the activity of the PFKFB4 promoter sensitized by overexpression of PPARγ^WT^. To evaluate whether the observed PPARγ-mediated induction of PFKFB4 expression was directly affect d by the transcriptional activity of its promoter, the binding of PPARγ with the PFKFB4 gene promoter was investigated by a chromatin immunoprecipitation (ChIP) assay. Four sequences in the human PFKFB4 promoter displayed significant homology with the PPARγ recognition motif (PPRE), AGGTCA-N-AGGTCA (upper panel of Figure 4F), which was predicted by PPRE Research (http://www.classicrus.com/PPRE/). Based on this prediction, we designed three pairs of primers for ChIP-PCR. The band from the ChIP-PCR pull-down by anti-PPARγ antibody revealed that PPARγ can bind the PFKFB4 promoter at site 1 and 2, which are close to each other and share one base pair (lower panel of Figure 4F). On the other side, using the PFKFB4 promoter at site 3 and 4, there is not significant alteration between the band of anti-PPARγ antibody and IgG control (Supplementary Figure S4D). Thus, our findings strongly support that PPARγ binds directly to the PFKFB4 promoter and increase its activity.

### Phosphorylation of PPARγ enhances glycolysis in HCC dependent on PFKFB4

**Figure 5:**
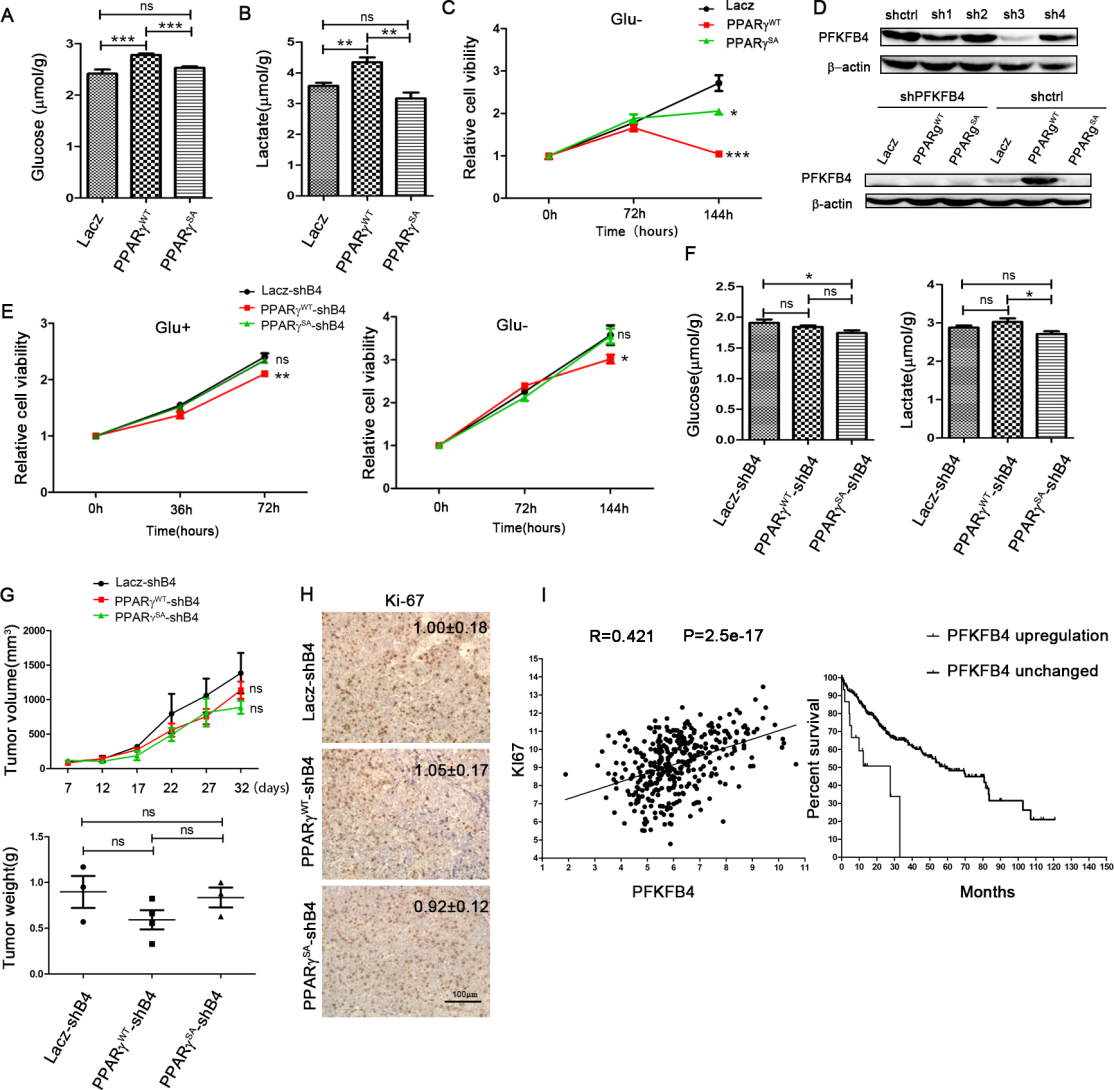
Phosphorylation of PPARγ enhances the glycolysis in HCC dependent on PFKFB4 Glucose consumption (**A**) and lactate production (**B**) in HepG2 overexpressed with PPARγ^WT^, PPARγ^SA^ or Lacz separately. (**C**) Growth curve of HepG2 overexpressed with PPARγ^WT^, PPARγ^SA^ or Lacz separately in the medium without glucose. (**D**) Knock down the PFKFB4 by shRNA in 293t overexpressed PFKFB4 (upper panel). Knock down the PFKFB4 in the HepG2 overexpressed with PPARγ^WT^, PPARγ^SA^ or Lacz.(down panel) (**E**) Growth curve of HepG2 overexpressed with PPARγ^WT^, PPARγ^SA^ or Lacz separately where PFKFB4 were knocked down in the medium with/without glucose. (**F**) After knocking down PFKFB4, Glucose consumption and lactate production in HepG2 overexpressed with PPARγ^WT^, PPARγ^SA^ or Lacz separately. (**G**) The volume and weight of tumors formed in nude mice using HepG2 cell line overexpressed with PPARγ^WT^, PPARγ^SA^ or Lacz separately whose PFKFB4 were knocked down. (**H**) Expression of Ki67 in these tumors by IH analysis. (**I**) The correlation analysis of Ki67 mRNA with mRNA levels of PFKFB4 (left) in HCC patients. (*n* = 373). Data represented the expression pattern in 373 patients of TCGA database. Kaplan–Meier graphs showing significant association of elevated PFKFB4 gene expression with shorter survival in a cohort of 373 HCC patients.

We identified that PPARγ phosphorylation can directly activate the expression of PFKFB4, which plays an important role in glycolysis. Therefore, we were interested in the influence of PPARγ phosphorylation on glycolysis. As shown in Figure 5A and 5B, overexpression of PPARγ^WT^ in cell lines increased glucose consumption by 11% and lactate production by 21% compared to the lacZ control, whereas there was no significant alternation as a result of overexpression of PPARγ^SA^ and Lacz. Furthermore, As shown in the Figure 5C, when glucose was excluded from the medium for 6days, the cells overexpressing PPARγ^WT^ reduced by the 60% compared to the cells overexpressing lacZ (*p* = 0.0009). Moreover, blocking PPARγ phosphorylation partially reversed the inhibition of proliferation. The result in Figure 5C suggest that the cells overexpressing PPARγ^WT^ are much more dependent on glucose than the other cells, which indicates that they utilized much more of the glucose in the medium. All these results indicate that phosphorylation of PPARγ enhances glucose utilization and glycolysis in HCC.

To investigate the key role of PFKFB4 in the influence of PPARγ phosphorylation on glycolysis and cell proliferation, we used shRNA to knockdown expression of PFKFB4 (Figure 5D). By measuring cell proliferation in the low-glucose medium (Figure 5E), we found that the difference in glucose sensitivity between PPARγ^WT^ and lacZ decreased significantly upon knockdown of PFKFB4. Moreover, upon knockdown of PFKFB4, PPARγ^WT^ cells grew slower than PPARγ^SA^ and lacZ cells in normal medium. Furthermore, we found that with knockdown of PFKFB4, there was no difference between PPARγ^WT^ and lacZ cells in glucose consumption and lactate production (Figure 5F). In a word, the effects of PPARγ phosphorylation on glycolysis and cell proliferation are dependent on PFKFB4 expression.

Next, we were interested in the functions of PFKFB4 *in vivo*. The three HepG2 cell lines, overexpressing PPARγ^WT^, PPARγ^SA^ and lacZ separately, were implanted into nude mice. Upon knockdown of PFKFB4, tumour volume and tumour weight were not up-regulated by overexpressing either PPARγ^WT^ or PPARγ^SA^ compared to LacZ (Figure 5G). Accordingly, the expression of Ki-67 protein was also unchanged among the tumours in which PFKFB4 was knocked down (Figure 5H). Herein, we identified that PFKFB4 expression is required for the promotion of cell proliferation by PPARγ phosphorylation *in vivo*.

To investigate this hypothesis further, we analysed the expression of effector cell proliferation marker (Ki67) and PFKFB4 mRNA within the tumours of 373 HCC patients (data obtained from The Cancer Genome Atlas [TCGA]). Interestingly, this showed that the amount of Ki67 mRNA positively correlated with PFKFB4 mRNA(r = 0.42, *p* < 0.001) (Figure 5I). Moreover, the patients who had elevated PFKFB4 gene expression (red line) had shorter survival rates than those without high PFKFB4 gene expression (blue line) (*p* = 0.0045). Taken together, these results support a key function of PFKFB4 in tumour growth and progression.

## DISCUSSION

Here, we show for the first time that PPARγ phosphorylation at Ser84/Ser82 (Ser112 in PPARγ2) occurs in the majority of HCC cases investigated and in liver tumours from human samples and a mouse model, whereas blockade of PPARγ phosphorylation by a kinase inhibitor or site-mutation both decrease the aggressiveness and tumourigenicity of hepatoma/hepatocarcinoma cells.

PPARγ is most famous for its roles in adipogenesis, in which it induces the expression of genes involved in lipid synthesis and adipocyte differentiation. Accumulating evidence has demonstrated the involvement of PPARγ in glucose metabolism as well. For example, the thiazolidinediones, PPARγ agonists, have been widely used for the treatment of type 2 diabetes mellitus owing to their effectiveness in lowering blood glucose levels [39]. Interestingly, it has been reported that PPARγ contributes to M2 isoform of pyruvate kinase (PKM2) and hexokinase 2 (HK2) expression, which catalyse specific reactions in glycolysis in fatty liver [40]. Similarly, our present findings identified that PFKFB4 is a novel PPARγ target gene, through which PPARγ may promote glycolysis and cell proliferation in HCC. In a word, PPARγ as a novel transcription factor turning on specific glycolytic isozymes that are frequently up-regulated in pathophysiological growth will be crucial to understanding the multi-faceted effects of PPARγ.

The paradoxical effects resulting from PPARγ activation are derived from a complex balance of the anti- versus pro-tumour functions of the PPARγ protein and its ligands in a given system. As expected, our *in vivo* study verified that DEN-induced HCC cannot be inhibited by a PPARγ agonist (RSG) alone because TZD drugs can exert protumourigenic actions in certain rodent models [40]. However, because PPARγ appears to be a tumour-type and tumour stage-specific modulator that is regulated by the ERK cascade, the MEK inhibitor PD0325901 together with RSG inhibited the growth of the tumours much better than PD0325901 along (Figure 2), which offers a potential opportunity for combination chemotherapy against HCC. Although no clinical evidence has been published on the combined use of ERK cascade inhibition and PPARγ activation in tumours [41], current therapeutic regimens inhibit the eicosanoid-mediated activation of the ERK cascade, and in conjunction with PPARγ activation, may provide a basis for differentiation-inducing therapy in combination with classical chemotherapeutics or biologics.

Consistently, our results indicated that phosphorylation of PPARγ up-regulated expression of PFKFB4 at the transcriptional level, suggesting that some “alternative” PPARγ transcriptional activity is increased under this condition. Because the site and kinase for PPARγ phosphorylation are not altered, the reason might lie in a specific PFKFB4 promoter that affects the binding between PPARγ and cis-acting elements upstream of the PFKFB4 gene. Furthermore, there is another possible explanation that phosphorylation of PPARγ results in this repressive transcription factor inactivation and thus activate/mobilize the target genes, such as PFKFB4. The exact mechanism remains to be elucidated in further studies.

In summary, our study provides the first evidence that the novel roles of PPARγ are linked to glycolysis by PFKFB4 for the regulation of glycolysis and proliferation in HCC cells. Deregulation of cellular metabolism is one of the hallmarks of cancer cells, and altered components of the metabolic pathway represent attractive therapeutic targets. Thus, the identification of the MEK/ERK/PPARγ/PFKB4 axis regulating glycolysis elicits a potentially new approach to targeting a tumour-specific metabolic pathway and understanding the mechanisms of hepatocarcinogenesis, its detection, therapeutic intervention, and prevention.

## MATERIALS AND METHODS

### Diethylnitrosamine (DEN)-induced hepatocellular carcinogenesis

Fourteen-day-old C57BL/6 mice were injected intraperitoneally (i.p.) with 25 mg/kg DEN (Sigma, St. Louis, MO, USA). Control mice were given an equivalent volume of saline. After 8 months, mice were euthanized, and their livers were collected and fixed in 10% formalin for histological and immunohistochemical analysis.

### Chromatin Immunoprecipitation

A total of 1 × 10^7^ HepG2 cells stably expressing PPARγ were crosslinked with 3.7% formaldehyde at room temperature for 10 min. Cells were incubated with 0.125 M glycine to terminate crosslinking and washed twice with PBS. DNA was prepared by using a SimpleChIP Enzymatic Chromatin IP Kit (Santa Cruz Biotechnology, CA, USA) according to the manufacturer's instructions. A DNA fragment encompassing the indicated region of the human PFKFB4 promoter was amplified using 35 cycles of PCR at 94°C for 30 s, 60°C for 30 s, and 72°C for 30 s with specific primers. All amplified products were resolved on 4% agarose gel [33].

### Microarray analysis

Total RNA was isolated from HepG2 cells stably expressing WT and S112A mutant of PPARγ. Array hybridization and scanning were performed using a NimbleGen Hybridization System and Axon GenePix 4000B microarray scanner. All array data were imported into NimbleScan software (version 2.5) for grid alignment and expression data analysis. Further analysis was performed using Agilent GeneSpring GX software (version 11.5). The raw data were uploaded as the supplementary data.

## SUPPLEMENTARY MATERIALS METHODS AND FIGURES


