# Supplementary material for: Phosphorylation of PPARγ at Ser84 promotes glycolysis and cell proliferation in hepatocellular carcinoma by targeting PFKFB4
Source: Oncotarget. 2016 Oct 19;7(47):76984–94. doi: 10.18632/oncotarget.12764 (PMC5363564; doi:10.18632/oncotarget.12764)
Supplement: Supplementary file 1 [file oncotarget-07-76984-s001.pdf]

Phosphorylation of PPARγ at Ser84 promotes glycolysis and cell proliferation in hepatocellular carcinoma by targeting PFKFB4

Supplementary Materials

MATERIALS AND METHODS

Ethics statement

The study was carried out strictly according to National Institutes of Health (NIH) Guidelines for Laboratory Animal Care and Guidelines for the welfare and use of animals in cancer research. The protocol was approved by the Committee on the Animal Care and Use Committee of Nanjing University. All efforts were made to minimize animal suffering and to reduce the number of animals used. For experiments involving the use of human tissue, written consent forms were signed by all the well-informed patients according to the ethical protocols of Nantong People's Hospital.

Patients and tissue samples

A total of 27 patients treated for HCC at Nantong Third People's Hospital (Nantong city, Jiangsu Province, China) in 2015 were enrolled in this study. None of the patients had received radiotherapy or chemotherapy before surgery. 27samples of HCC and adjacent normal tissues were collected. The clinical stage of HCC was evaluated according to TNM system. Pathological TNM staging was performed according to the criteria of the American Joint Committee on Cancer. Tissue samples were fixed in 10%formalin within 1h after surgical removal, embedded in paraffin, and sectioned consecutively at 4mm thickness using a rotary microtome for immunohistochemical assays.

Cell culture

HepG2, Hep3B, SMMC-7221, Hepa1-6, and HEK293T cells were purchased from Shanghai Institutes for Biological Sciences, Chinese Academy of Sciences (Shanghai, China) and grown in Dulbecco's modified Eagle's medium (DMEM) with 10% FBS and antibiotics. 293FT cells were purchased from Invitrogen (Carlsbad, CA, USA) and maintained in complete medium containing 500 mg/mL geneticin (Merck, Darmstadt, Germany). All cells were maintained at 37°C with 5% CO_2 atmosphere in a humidified incubator.

Immunofluorescence

Cells were rinsed in ice-cold PBS followed by 15 min incubation with 4% paraformaldehyde at RT. Cells were then briefly rinsed with PBS, incubated for 5 min with 0.2% Triton X-100, and washed 3 × 5 min with PBS. Nonspecific binding sites were blocked with 5% BSA for 1 hour at RT. The primary antibody anti-PPARγ (Cell Signaling Technology, MA, USA) was diluted to 1:200 and incubated overnight at 4°C. Following incubation, the cells were washed 3 × 5 min with PBST and incubated with Cy3-conjugated secondary antibody (Beyotime, Jiangsu, China) for 1 hour at RT. Nuclei staining was performed by incubating the cells with DAPI (Beyotime, Jiangsu, China) before mounting the processed coverslips onto ethanol-cleaned glass slides using Dabco mountant. The specimens were viewed with an inverted confocal microscope (Olympus, Japan).

Western blot

Cell samples were lysed with radioimmunoprecipitation assay lysis buffer ((Beyotime, Jiangsu, China) containing 100 mM phenylmethylsulfonyl fluoride, and the protein concentrations were determined using a BCA kit (Beyotime, Jiangsu, China). The whole cell lysate was subjected to reducing 10% SDS-polyacrylamide gel electrophoresis (PAGE), transferred onto PVDF membranes (Millipore, Bedford, MA, USA), blocked with 5% skim milk for 1 hour at room temperature, and immunoblotted at 4°C overnight with the primary antibody for PPARγ (Cell Signaling Technology, MA, USA), pPPARγ (Abcam, Cambridge, UK), PCNA (Cell Signaling Technology, MA, USA), ERK, pERK, MEK and pMEK (Bioworld, MN, USA). Secondary antibody binding was visualized using an Enhanced Chemiluminescent Method Kit (Cell Signaling Technology, MA, USA). As an internal control for equal protein loading, blots were stripped and probed with antibodies against GAPDH or β-actin (Kangchen, China).

Real time PCR

Total RNA was extracted from cells using Trizol reagent (Invitrogen, Carlsbad, CA, USA) according to the manufacturer's instructions. RNA concentration was measured spectrophotometrically by absorbance at 260 nm, and 2 mg of RNA was reverse-transcribed into cDNA. The reverse transcriptional reaction was carried out at 50°C for 60 min and 70°C for 10 min. Real-time PCR quantification was performed following standard protocols using SsoFast EvaGreen Supermix (Bio-Rad, USA) on the CFX96 Real-Time System C1000 Thermal Cycler (Bio-

Rad, USA). The gene expression levels for each amplicon were calculated using the $\Delta\Delta C_T$ method and normalized against GAPDH mRNA.

Cell viability assay

Cell viability was determined by 3-(4,5-dimethylthiazol-2-yl)-2,5-diphenyltetrazolium bromide (MTT) (Sangon, Shanghai, China) assay. Briefly, cells (4×10^3/ well) were seeded in 96-well plates and treated with or without rosiglitazone. After 24 h treatment, MTT reagent was added to the media at a final concentration of 0.5 mg/mL. Four hours later, formazan crystals generated by cellular reduction of the MTT reagent were dissolved in dimethylsulfoxide (DMSO) for 10 min at 37°C, and the absorbance was determined at a wavelength of 490 nm using an ELISA reader (Safire, Tecan, AG, Switzerland).

Lentivirus production and transduction

Point mutations of PPARγ constructs were generated by using a Muta-direct™ Site-Directed Mutagenesis Kit (SBS Genetech, Shanghai, China). Lentiviruses were produced in 293FT cells with a packaging mix (ViraPower™ Lentiviral Expression Systems, Invitrogen, Carlsbad, CA). Lentiviruses were concentrated by using PEG-it™ Virus Precipitation Solution (System Biosciences Mountain View, California, USA), and the viral titre was determined by serial dilution.

Clonogenic assay

Cells were harvested and re-suspended in complete growth media. Then, 500 cells were seeded onto six-well plates and allowed to grow until visible colonies formed (14 days) (1). Cell colonies were fixed with 4% paraformaldehyde solution for 15 minutes and stained with 0.4% (w/v) Giemsa dye (in 70% methanol) for 60 minutes. Colonies were counted digitally using ImageJ software.

Histology and Immunohistochemistry

Formalin-fixed, paraffin-embedded samples were sectioned and stained with haematoxylin and eosin (H&E). The expression and localization of various proteins in liver tissues were assessed by immunohistochemical staining of tissue sections with antibodies against Ki67 (Boster, Wuhan, China), pMEK and pERK (Bioworld, MN, USA), pPPARγ (Abcam, Cambridge, UK). Negative controls were obtained by omitting the primary antibody.

Luciferase assay

Cells at approximately 80% confluence in 24-well plates were transiently transfected using Lipofectamine 2000 transfection reagent (Invitrogen, Carlsbad, CA) with a plasmid containing the luciferase gene under the control of three tandem PPAR-response elements (3× PPRE TK-luciferase). The *Renilla* luciferase control reporter vector (pRL-TK) was used as an internal control. 12 hrs after transfection, cells were serum-deprived (1% FBS) and then treated with rosiglitazone for an additional 12 h. Cells were then lysed, and their luciferase activities were measured using a Dual-Luciferase Reporter System (Promega, Madison, WI, USA).

Electrophoretic mobility shift assay (EMSA)

EMSA was performed as previously described (2). Nuclear extracts were prepared using a nuclear and cytoplasmic protein extraction kit (Beyotime, China). The oligonucleotides used were CAAAACTAGGTCAAAGGTCA and its complementary sequence. The biotin-labelled complementary oligonucleotides were mixed in annealing buffer and incubated at 95°C for 10 min followed by cooling to room temperature for 1 h. EMSA was performed using a LightShift Chemiluminescent EMSA kit (Thermo Fisher Scientific, Waltham, MA, USA). Biotin end-labelled DNA containing the binding site of interest was incubated with nuclear extracts. The mixture was electrophoresed in 4% polyacrylamide gels and then transferred to a nylon membrane (Roche Applied Science). Staining and detection procedures were carried out according to the kit protocol.

Glucose consumption, lactate secretion

Glucose and lactate concentrations in media incubated with cells were determined by the use of glucose and lactate assay kits from JiangChen (NanJing, China). Fresh medium was added to a 6-well plate of cells and analysed after 24 hr (triplicate samples). Total protein content was used for normalization (3).

Xenograft transplantation

All experimental procedures involving animals were approved by the Animal Care and Use Committee of Nanjing University, China. Tumour xenografts were established by subcutaneous injection of 2×10^6 HepG2 cells into the left flanks of 4-week-old BALB/c nude mice. Tumour length (L) and width (W) were measured with callipers, and tumour volume was estimated as $V = (L \times W^2)/2$. Mice were euthanized at 6 weeks after injection, and tumour weights were measured.

Image analysis

The intensity of the staining signal was measured and documented using Image-Pro Plus 6.0 image analysis software (Media Cybernetics, Inc. Silver Spring, MD USA)(5). The mean densitometry of the digital image (200×) was designated as representative protein staining intensity (indicating relative protein expression level). The signal density of tissue areas from six randomly selected images were counted blindly and subjected to statistical analysis.

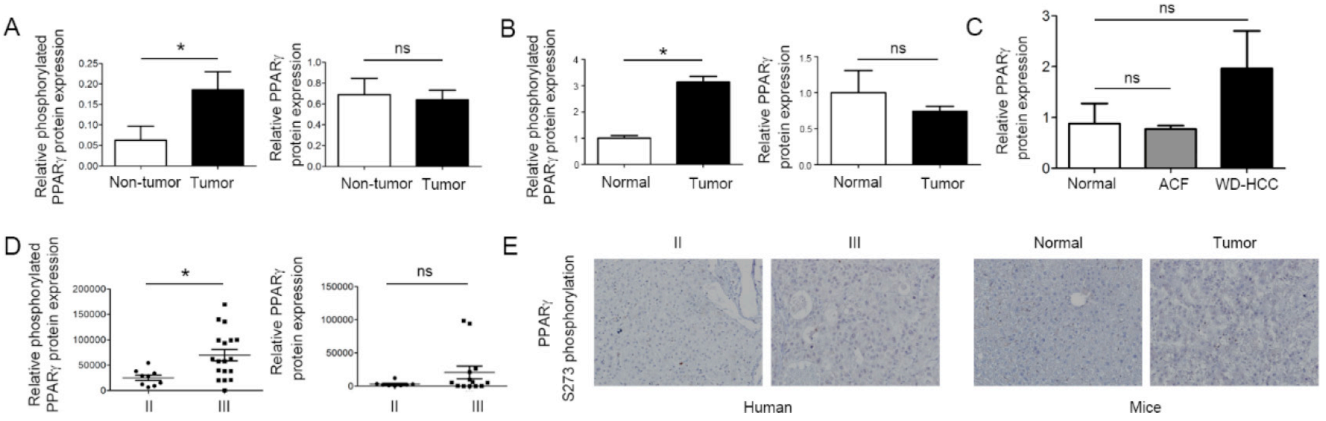

Supplementary Figure S1: (**A**) Quantification of relative phosphorylated PPARγ and PPARγ in Figure 1A. (**B**) Quantification of relative phosphorylated PPARγ and PPARγ in figure 1B (mice sample). (**C**) Quantification of relative PPARγ in Figure 1C. (**D**) Quantification of relative phosphorylated PPARγ and PPARγ in figure 1E (E) PPARγ phosphorylation in Ser273 between the normal and tumor tissue from the mice liver and between phase II and III tumour from human liver.

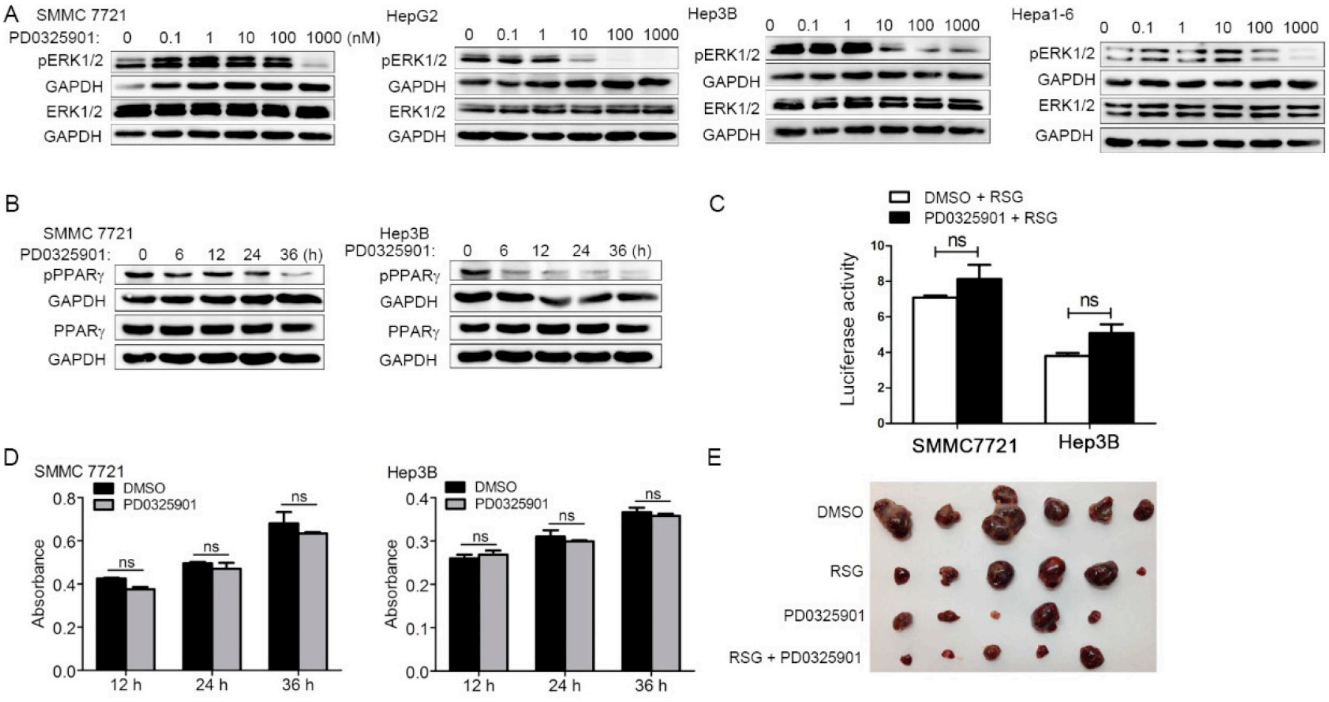

Supplementary Figure S2: (**A**) the expression of p-ERK and ERK in different cell lines treated with indicated PD325901 concentration (**B**) the expression of p-PPARγ and PPARγ in SMMC7721 and Hep3B treated with PD325901 for 0, 6, 12, 24, 36 hrs separately. (**C**) PPARγ transcriptional activity was measured in 7721 and Hep3B by luciferase reporter gene assay with/without PD0325901. (**D**) Proliferation of SMMC7721 and Hep3B treated with or without PD0325901. (**E**) Tumors from the mice treated with DMSO, RSG only, PD0325901 only, or RGS+ PD0325901.

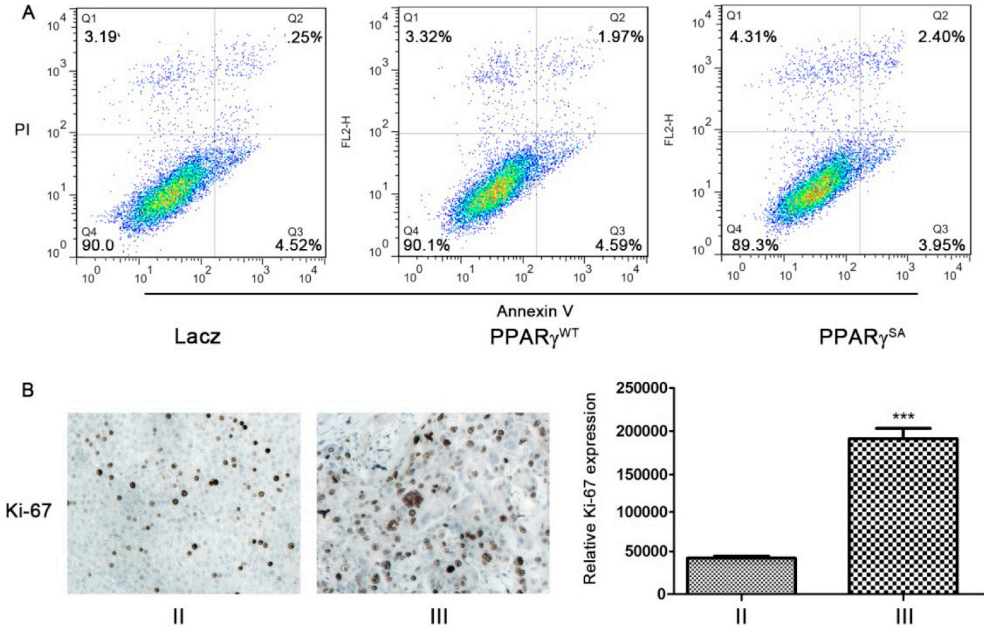

Supplementary Figure S3: (**A**) Apoptosis analysis of HepG2 stablly overexpressing PPARγWT, PPARγS112A or lacZ by PI/annexin V. (**B**) Expression of Ki67 in human phase II and phase III liver tumor by IHC analysis.

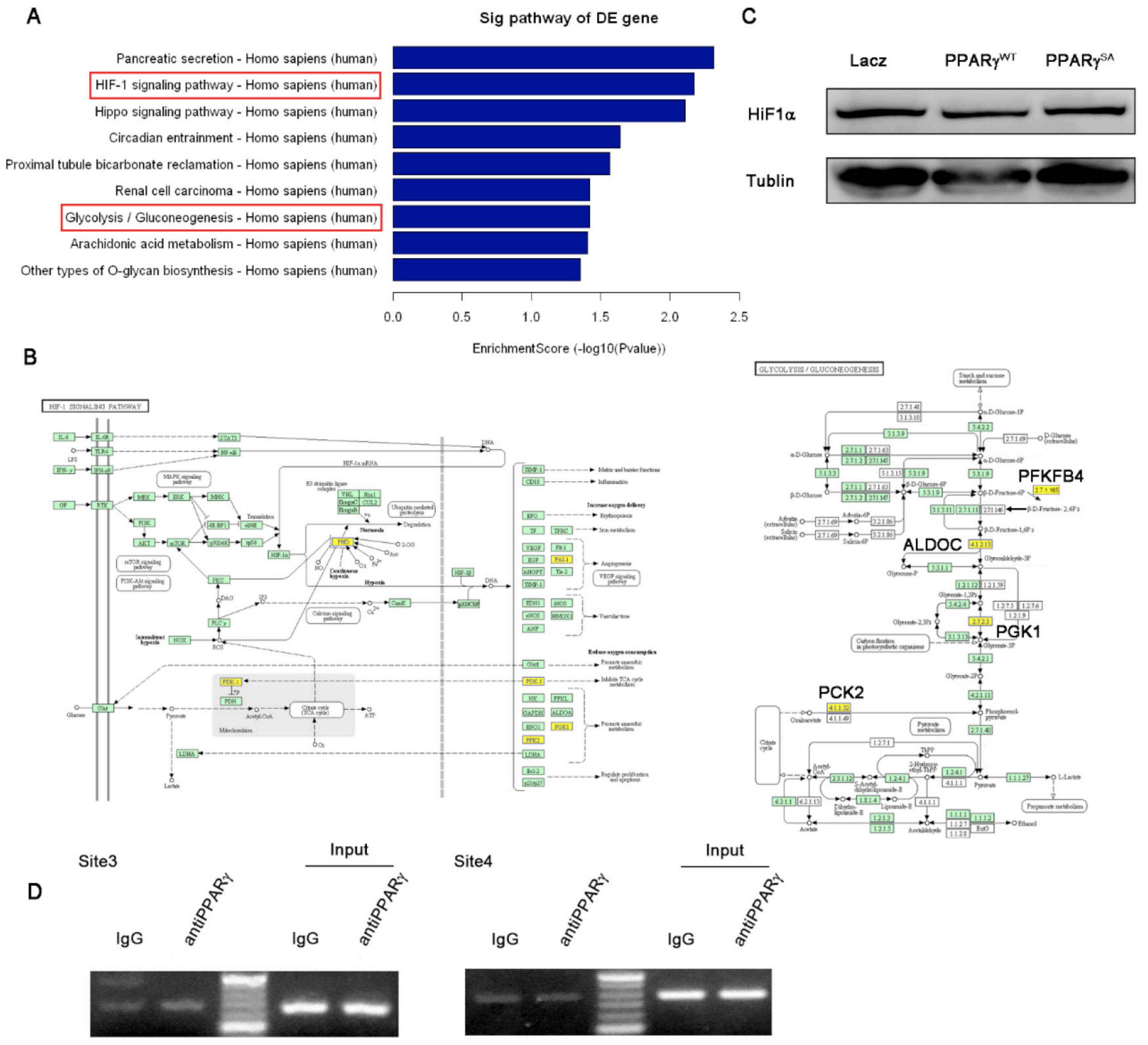

Supplementary Figure S4: (**A**) Significant difference in signal pathways between the HepG2 cells transfected with PPARγ^{WT} or PPARγ^{SA} (data from Gene-expression chips) (**B**) HIF-1 pathway and glycolysis pathway (yellow=significant down-regulation; green= no significant difference; white = no data) (**C**) HIF1α protein expression in HepG2 cells transfected with lacZ, PPARγ^{WT} or PPARγ^{SA}. (**D**) HepG2 was harvested for ChIP analysis with an anti-PPARγ antibody or preimmune IgG using site3 and site4 chip primers. Specific primers were used to amplify four sequences of the sites. Input chromatin was diluted to 1:1000. All PCR products were resolved by 2% agarose electrophoresis.
